# New Dipyrroloquinones from a Plant-Derived Endophytic Fungus *Talaromyces* sp.

**DOI:** 10.3390/molecules28237847

**Published:** 2023-11-29

**Authors:** Dandan Zhang, Xiaoqing Wang, Bo Liu, Shuhui Li, Yanlei Wang, Tao Guo, Yi Sun

**Affiliations:** 1 Institute of Chinese Materia Medica, China Academy of Chinese Medical Sciences, Beijing 100010, China; z2531817909@163.com (D.Z.); xiaoqw1307@outlook.com (X.W.); lubov_lb@126.com (B.L.); l1744140302@163.com (S.L.); w15739539626@163.com (Y.W.); 2 Henan Engineering Research Center of Medicinal and Edible Chinese Medicine Technology, Henan University of Chinese Medicine, Zhengzhou 450046, China; gt010010@163.com

**Keywords:** *Talaromyces* sp., endophytic fungus, dipyrroloquinone, anti-inflammatory, cytotoxic activity

## Abstract

Two new dipyrroloquinones, namely talaroterreusinones A (**1**) and B (**2**), together with four known secondary metabolites, terreusinone A (**3**), penicillixanthone A (**4**), isorhodoptilometrin (**5**), and chrysomutanin (**6**), were isolated from the solid culture of the endophytic fungus *Talaromyces* sp. by integrating mass spectrometry-based metabolic profiling and a bioassay-guided method. Their planar structures and stereochemistry were elucidated by comprehensive spectroscopic analysis including NMR and MS. The absolute configuration at C-1″ of terreusinone A (**1**) was established by applying the modified Mosher’s method. Compounds **1**–**6** were evaluated for anti-inflammatory activity and cytotoxicity. As a result, **1**–**3** inhibited the LPS-stimulated NO production in macrophage RAW264.7 cells, with IC_50_ values of 20.3, 30.7, and 20.6 µM, respectively. Penicillixanthone A (**4**) exhibited potent cytotoxic activity against Hep G2 and A549 cell lines, with IC_50_ values of 117 nM and 212 nM, respectively, and displayed significant antitumour effects in A549 cells by inhibiting the PI3K-Akt-mTOR signalling pathway.

## 1. Introduction

Dipyrroloquinones are unusual alkaloids in nature, whose basic skeleton possesses a highly symmetrical pyrrole-benzoquinone-pyrrole tricyclic system [1]. Currently reported dipyrroloquinones have three types of structures, including terreusinones, zyzzyanones, and tsitsikammamines, and these analogues are mainly from marine sponges and fungi [2,3,4,5]. These compounds are biosynthetically derived from seco-pyrroloiminoquinones such as makaluvamines and discorhabdins [6]. Due to the highly conjugated system, terreusinones A-C had a significant UV-A protective ability and also exhibited high inhibitory activity against tyrosine phosphatases and antioxidant activity [7,8]. The bioactive dipyrroloquinones were synthesized since some of the seco-pyrroloiminoquinones displayed potent cytotoxicities against the tumour cell lines [9]. In this paper, the dipyrroloquinones were discovered from the *Talaromyces* genus for the first time, and it was certified that this type of dipyrroloquinones exhibited moderate anti-inflammatory activity in LPS-induced RAW264.7 cell lines.

The *Talaromyces* genus has been studied in the classification framework of Penicillium which can produce soft-wall ascomata. The genus includes about 171 species, widely existing in soil, plants, and sponges [10,11]. The genus *Talaromyces* can produce abundant bioactive secondary metabolites, including polyketones, alkaloids, quinones, steroids, esters, terpenoids, and coumarins. These compounds displayed various biological activities such as antitumor and antibacterial effects, and so on. Several *Talaromyces* species have been certified to be effective biocontrol agents against pathogens in soil [12,13,14,15]. However, some species of *Talaromyces* produced typical hepatoxic and nephrotoxic mycotoxins, such as luteoskyrin, cyclochlorotine, erythroslkyrine, and islanditoxin.

**Figure 1:**
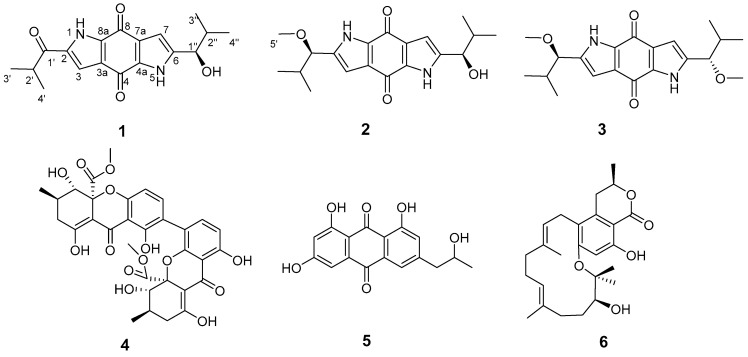
Structures of compounds **1**–**6**.

As a part of our ongoing studies on the bioactive secondary metabolites from endophytic microbes, we assembled a library of more than 600 endophytes from medicinal plants of different environments [16,17]. Using a global natural product social (GNPS) molecular networking analysis, compounds can be grouped according to MS/MS fragmentation patterns [16,17,18,19,20]. It revealed that some fungi in our library could produce chemically diverse secondary metabolites. Our chemical investigation led to the discovery of two new dipyrroloquinones, talaroterreusinones A (**1**) and B (**2**), together with four known secondary metabolites including terreusinone D (**3**) [3], penicillixanthone A (**4**) [21], isorhodoptilometrin (**5**) [22], and chrysomutanin (**6**) [23] from the *Talaromyces* sp. of the medicinal plant *Stellera chamaejasme* Linn in Figure 1. This study describes the molecular networking analysis of the extract, isolation and structural elucidation of the new compounds, as well as the evaluation of the cytotoxic activities against Hep G2 and A549 tumour cells and inhibitory effects on LPS-induced NO production in macrophage RAW264.7 cells.

## 2. Results and Discussion

### 2.1. Molecular Networking-Based Prioritization of the Isolation Workflow

In order to prioritize the isolation of dipyrroloquinones, the total secondary metabolites of *Talaromyces* sp. were determined by UPLC-Q-TOF-MS/MS. The MS^E^ data was processed using the GNPS web platform and analysed by the feature-based molecular networking strategy. The data were subsequently exported via Cytoscape 3.8.2 software to construct the Molecular Networking (MN).

**Figure 2:**
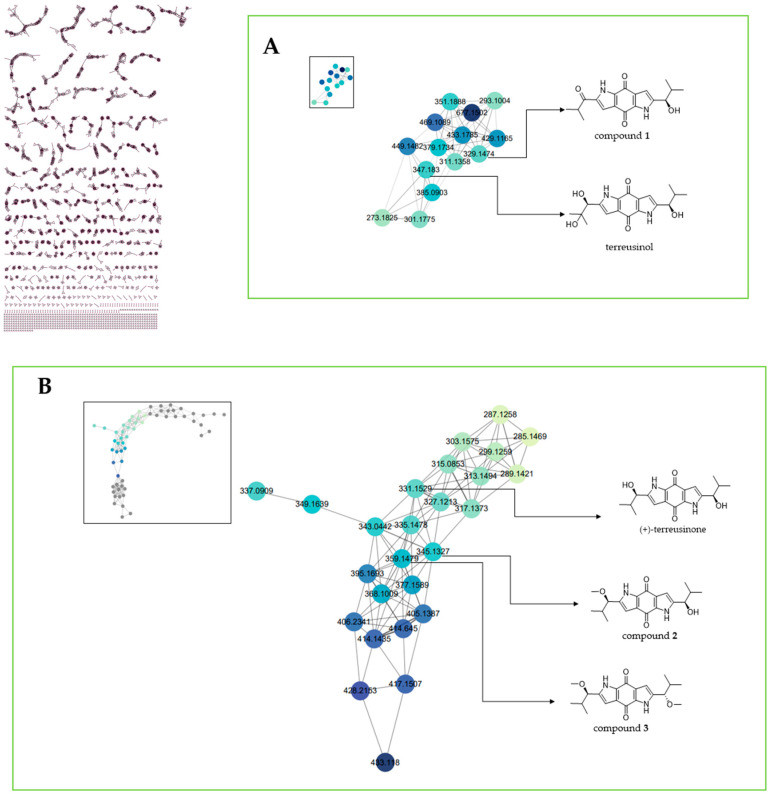
Partial molecular network using MS^E^ data of the total secondary metabolites, according to the legend of the *m*/*z* size of the responsible metabolites. (**A**) A compound structurally relates to terreusinol in the cluster. (**B**) Two compounds structurally relate to terreusinone in the cluster.

As depicted in Figure 2A, the MS^E^ data of the total secondary metabolites were presented in purple circles, and the annotated nodes were displayed as green and blue ones. All precursor ions formed a molecular network with 348 clusters and 4302 nodes. The annotated network displayed the presence of two clusters composed of dipyrroloquinone masses. In one of the clusters (Figure 2A), the structurally related dipyrroloquinone of terreusinol (*m*/*z* = 347.183) was connected to the nodes *m*/*z* 329.1474 and 379.1743, which were supposed to be new dipyrroloquinones. In the other cluster (Figure 2B), the structurally related dipyrroloquinones of terreusinone D (compound **3**, *m*/*z* 359.148) and (+)-terreusinone (*m*/*z* 331.153) were directly connected to an additional node, which indicated the presence of a new putative analogue at *m*/*z* 345.133. Consequently, the nodes of dipyrroloquinone analogues were targeted for isolation and structural determination. 

### 2.2. Structural Identification

The molecular formula of talaroterreusinone A (**1**) was assigned as C_18_H_20_N_2_O_4_ based on the HRESIMS data (Figure S1), suggesting ten degrees of unsaturation. The ^1^H NMR spectrum data (Figure S2) of **1** displayed the signals of two olefinic protons at *δ*_H_ 7.31 (s, H-3) and 6.38 (s, H-7), one oxygenated methine proton at *δ*_H_ 4.27 (dd, *J* = 5.1, 6.6 Hz, H-1″), and four methyls at *δ*_H_ 1.09 (d, *J* = 6.8 Hz, H_3_-3′, 4′), 0.86 (d, *J* = 6.7 Hz, CH_3_-3″), and 0.77 (d, *J* = 6.8 Hz, CH_3_-4″). Its ^13^C NMR (Figure S3) and HSQC (Figure S4) spectra revealed the presence of eighteen carbons, including three carbonyls, six quaternary carbons, five methines, and four methyls (Table 1). 

**Figure 3:**
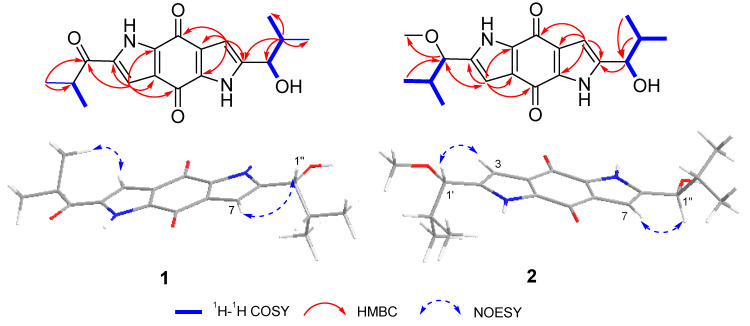
Key ^1^H-^1^H COSY, HMBC and NOESY correlations of **1** and **2**.

Further comprehensive analysis of the 2D NMR data (Figures S4–S7) confirmed the whole structure of **1**, which had a dipyrroloquinone skeleton and resembled that of terreusinone [2] (Figure 3). The HMBC correlations from H-7 to C-6, C-8, C-7a and C-4a, as well as from H-3 to C-1′, C-2, C-3a, C-4, and C-8a, established the structural skeleton of dipyrrolobenzoquinone. The proton spin systems of H-1″/H-2″/H-3″/H-4″ and H-1″/1″-OH, coupled with the HMBC correlations from 1″-OH and H-1″ to C-6 and C-2″, and from H-1″ to C-7, revealed the connection between the pyrrole ring and the aliphatic chain, and the hydroxyl group at C-1″. Further HMBC correlations from H-3 and H-2′ to C-1′ confirmed the linkage of the ketone group and the pyrrole ring at C-2. Additionally, HMBC correlations from H-2′ to C-1′, C-3′, and C-4′ completed the establishment of the planar structure of **1**. The key NOESY cross-peaks of H-7 with H-1″ indicated that the hydroxyl group was *trans*-orientated with H-7. 

**Figure 4:**
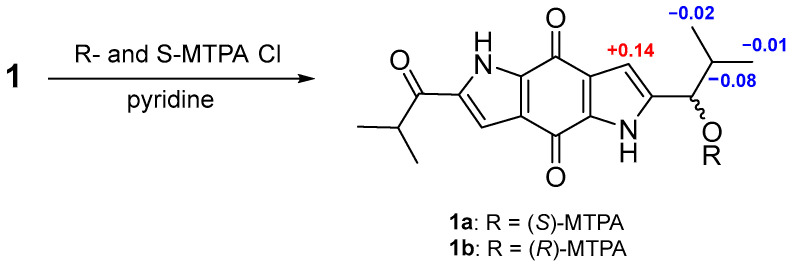
Values of Δ*δ*_S_ − *δ*_R_ of the MTPA esters in **1**.

To resolve the absolute configuration of C-1″, the modified Mosher’s derivatisation was carried out. Compound **1** was esterified with *R*-(−)- and *S*-(+)-α-methoxy-α-(trifluoromethyl) phenyl acetyl chloride (MTPA-Cl) to obtain the corresponding *S*- and *R*-MTPA esters (**1a** and **1b,** their HRESIMS data were showed in Figures S8 and S9), respectively. Both esters’ chemical shifts in the proximity of C-1″ were determined by the ^1^H NMR and ^1^H-^1^H COSY (Figures S10–S13) analysis and confirmed the absolute configuration of *R* in C-1″ by calculating the Δδ*_S–R_* values (Figure 4). 

Talaroterreusinone B (**2**) was determined to be C_19_H_24_N_2_O_4_ according to the ^13^C NMR data and HRESIMS (Figure S14) data *m*/*z* 345.1791 [M + H]^+^, indicating ten degrees of unsaturation. Analysis of the ^1^H and ^13^C NMR data (Figures S15 and S16) suggested that **2** was a dipyrroloquinone analogue of **1** (Table 1). Compared to its ^13^C NMR data with that of **1**, it displayed the presence of one methoxyl group, which was further supported by the HMBC (Figure S17) correlations from H-1′ to C-2, C-3 and C-2′. It indicated that the ketone at C-1′ in **1** was replaced by a methoxyl group in **2**. Furthermore, the key NOESY (Figure S18) cross-peaks of H-7 with H-1″ and H-3 with H-1′ also revealed a *trans* configuration (Figure 4). Thus, combine with other 2D NMR data (Figures S19 and S20), compound **2** was elucidated as the 1′-methoxy analogue of **1** and named talaroterreusinone B.

All compounds were evaluated for their cytotoxicity (Table 2). Among the tested compounds, penicillixanthone A (**4**) had significant activity against A549 and Hep G2 tumour cell lines, with IC_50_ values of 0.117 and 0.212 µM, respectively. Compounds **1**–**3** and **5**–**6** showed weak cytotoxicity with IC_50_ values of more than 50 µM against A549 and HepG2 cells (Figures S21 and S22). On the other hand, compounds **1**, **2**, and **3** displayed moderate inhibitory activity against the LPS-induced NO production in the macrophage RAW264.7 cell line, with IC_50_ values of 20.3, 30.7, and 20.6 µM, respectively (Table 3).

**Figure 5:**
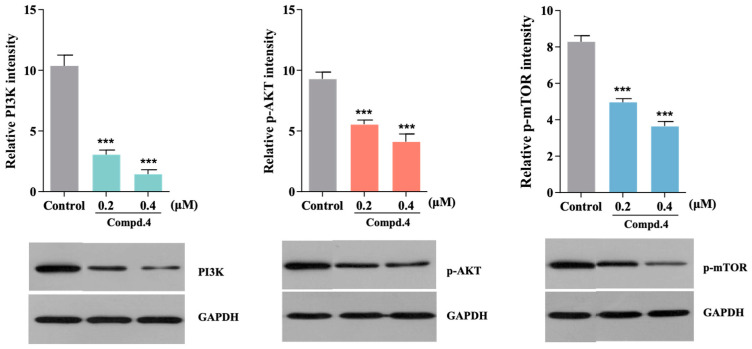
The protein expression of the PI3K-Akt-mTOR signalling pathway of **4** was evaluated by western blot analysis. A549 cells were treated with **4** (0.2 and 0.4 μM) for 24 h, and the expression levels of PI3K, Akt and mTOR were tested by the responsible antibodies. Compound **4** inhibited the expression of PI3K, Akt and mTOR after treatment. Data are represented as the mean ± SD, *n* = 3, *** *p* < 0.01 vs. Control.

Considering the significant cytotoxicities of penicillixanthone A (**4**), we evaluated its plausible molecular mechanism in A549 tumour cells using western blot analysis in Figure 5. Compound **4** (0.2 and 0.4 µM) was firstly pretreated for 24 h to test the PI3K expression. As a result, **4** could decrease the phospho-PI3K expression after intervention at the concentrations of 0.2 μM and 0.4 μM, respectively. Additionally, phospho-mTOR/Akt was also significantly suppressed after the intervention of **4** at the above same concentrations. Thus, it was demonstrated that **4** played a potential role in the regulation of the PI3K-Akt-mTOR signalling pathway.

## 3. Materials and Methods

### 3.1. General Experimental Procedures

NMR spectra were determined on a Bruker ARX-600 spectrometer (Bruker, Karlsruhe, Germany) operating at 600 MHz for ^1^H and 150 MHz for ^13^C, using DMSO-*d*_6_ (*δ*_H_ 2.50 and *δ*_C_ 39.50) as residual solvent. HRESIMS were measured on a Waters Vion QTOF/MS (Waters Mocromass, Manchester, UK) in positive electrospray ionization mode. Column chromatography (CC) was carried out on silica gel (200–300 mesh, Qingdao Marine Chemical Factory, Qingdao, China), and octadecyl-functionalized silica gel (ODS) (50 µm, YMC, Kyoto, Japan). Thin-layer chromatography (TLC) was carried out with glass-precoated silica gel GF254 plates (Qingdao Marine Chemical Factory). Semi-preparative HPLC was performed on the Agilent 1260 system using Cosmosil 5C_18_-ARII columns (4.6 mm × 250 mm, 5 µm, Kyoto, Japan) and the detection wavelengths were 280 and 320 nm.

### 3.2. Fungal Material

The fungus strain *Talaromyces* sp. in this work was isolated from a fresh blade of the plant *Stellera chamaejasme* Linn, collected from Nei meng gu province, China. 

The tender leaves of *Stellera chamaejasme* Linn were first washed with running tap water, then soaked in a 3.5% NaClO solution and 75% ethanol successively, and finally dried on the plant surface. The leaves were sliced with a sterilized blade, and the internal tissues were exposed to a potato dextrose agar (PDA) medium. The dishes were cultured at 27 ℃ and observed daily. The mycelia of the emerging colonies were re-inoculated to the fresh PDA dishes until pure cultures were afforded.

A BLAST search result showed that the sequence was the most similar (98.3%) to the sequence of *Talaromyces* sp. (compared to MH861710.1). The strain is currently stored in the Institute of Chinese Materia Medica, China Academy of Chinese Medical Sciences.

### 3.3. Fermentation and Isolation

The (PDA) with *Talaromyces* sp. was cut into small pieces and then inoculated into 120 sterilized Erlenmeyer flasks containing 40 g of rice in 60 mL of distilled water. After, the flasks were cultured at 27 °C for 14 days, and the solid cultures were extracted by ethyl acetate three times and were then concentrated. The EtOAc layer was finally dissolved in methanol and extracted with petroleum ether (1:1) to get a crude extract (15 g). 

The crude extract was isolated by a flash ODS column with a gradient of MeOH-H_2_O (40:60, 60:40, 80:20 to 100:0) to yield four fractions (Fractions 1–4). Fraction 3 was then subjected to a silica gel column by gradient elution with PE/acetone from 40:1 to 0:1 and isolated four subfractions (3.1–3.4). Subfraction 3.2 was purified by reversed-phase HPLC using a gradient of 50 to 75% acetonitrile in H_2_O with 0.2% AcOH to yield **1** (2 mg, t_R_ 24 min) and **2** (3 mg, t_R_ 36 min). Subfraction 3.3 was also purified by reversed-phase HPLC by using 65% acetonitrile in H_2_O with 0.2% AcOH to get **3** (3 mg, t_R_ 17 min). Additionally, fraction 4 was subjected to a silica gel column by a gradient elution with CH_2_Cl_2_/MeOH (from 100:1 to 0:1) to get five subfractions (subfractions 4.1–4.5). Subfraction 4.3 was then purified by HPLC using a gradient solvent system from 45% to 60% acetonitrile in H_2_O with 0.2% AcOH to yield **4** (6 mg, t_R_ 20 min), **5** (4 mg, t_R_ 43 min), and **6** (3 mg, t_R_ 25 min).

Talaterreusinone A (**1**): yellow amorphous solid, ^1^H NMR (DMSO-*d*_6_, 600 MHz) and ^13^C NMR (DMSO-*d*_6_, 150 MHz) data (see Table 1); HR-ESI-MS *m*/*z* 329.1253 [M + H]^+^ (calculated for C_18_H_20_N_2_O_4_, 329.3733).

Talaterreusinone B (**2**): yellow amorphous solid, ^1^H NMR (DMSO-*d*_6_, 600 MHz) and ^13^C NMR (DMSO-*d*_6_, 150 MHz) data (see Table 1); HR-ESI-MS *m*/*z* 345.1791 [M + H]^+^ (calculated for C_19_H_24_N_2_O_4,_ 345.4159).

### 3.4. Mosher’s Derivatization Method

Compound **1** (0.5 mg) was dissolved in an anhydrous mixture of pyridine and dichloromethane (0.25 mL) in a 2 mL centrifuge tube, then separately with (R)-MPTACl and (S)-MPTACl (5.0 µL) and magnetic stirrers to cause a reaction (room temperature, 12 h) [24]. After the reaction stopped, it was extracted with dichloromethane and water three times. The organic fractions were concentrated and purified by HPLC. Finally, 0.2 mg of **1a** was obtained. In the same manner, 0.2 mg of **1b** was prepared. 

### 3.5. Cytotoxic Activity Assay

The 3-(4,5-dimethyl-2-thiazolyl)-2,5-diphenyl-2-h-tetrazole ammonium bromide (MTT) experiment was applied to test the cytotoxic activity for **1**–**6** against human lung cancer cells A549 and liver cancer cells Hep G2. Cells in RPMI-1640 medium (10% FBS, 0.4% penicillin–streptomycin solution) were digested with trypsin and diluted to the concentration of 1 × 10^4^ cells/mL. The cell suspensions were diluted and added into a 96-well microtiter plate with 200 µL per well. The plate was then incubated in a CO_2_ incubator at 37 °C. Compounds **1**–**6** were added to the plate wells after 24 h and incubated for 72 h. Adriamycin (ADM) was used as the positive control, and DMSO as the blank control. The MTT solution was added after 72 h incubation, followed by incubation for 4 h. The cells were successively disrupted with 200 µL of DMSO for 10 min after the supernatant liquid was removed. The absorption was measured at 570 nm [25].

### 3.6. Anti-Inflammatory Activity Assay

RAW264.7 cells were cultured in DMEM with 10% fetal bovine serum (FBS) and 1% penicillin-streptomycin at 37 °C in 5% CO_2_. The MTT assay was used to evaluate cell viability. RAW264.7 cells were seeded on 96-well plates at 5 × 10^5^ cells/well [26]. After incubation with compounds **1**–**6** at five concentrations (20, 10, 5, 1, and 0.2 mg/mL) for 24 h, cells were treated with MTT (5 mg/mL) at 37 °C for 4 h. Then, the supernatant was removed and the formalin was crystalised with DMSO. The absorbance was measured at 570 nm. The Griess reaction was applied to measure the accumulation of NO_2_^−^ in culture supernatant. RAW264.7 cells were treated with the compounds for 1 h then with the presence of LPS (1 μg/mL) for 24 h. The culture supernatant fluids (50 µL) and Griess reagent (50 µL) were mixed and incubated at room temperature. The absorbance was read at 570 nm after 15 min.

## 4. Conclusions

A chemical study of the endophytic fungus *Talaromyces* sp. led to the isolation and identification of two novel dipyrroloquinones, talaroterreusinones A (**1**) and B (**2**), and four known compounds, terreusinone D (**3**), penicillixanthone A (**4**), isorhodoptilometrin (**5**), and chrysomutanin (**6**). Compounds **1** and **2** were identified by comprehensive spectroscopic analysis and Mosher’s method. To our knowledge, this is the first time discovery of dipyrroloquinones from the fungal genus *Talaromyces*. The new dipyrroloquinones had a highly symmetric pyrrolio-benzoquinone-pyrrole tricyclic system, which is rare from microbial origin. All compounds were evaluated for their cytotoxic activity and anti-inflammatory activity. The anti-inflammatory effect of LPS-induced NO production in RAW264.7 cells showed that the dipyrroloquinones (**1**–**3**) possessed moderate activity, with the EC_50_ values between 20.3 and 30.7 µM. Penicillixanthone A (**4**) exhibited potent cytotoxic activity against both Hep G2 and A549 tumour cells, and it regulated the expression of the PI3k-Akt-mTOR signalling pathway. However, the three dipyrroloquinones didn’t exhibit strong cytotoxicity against Hep G2 and A549 tumour cells. Thus, we isolated two new dipyrroloquinones which have been already reported with less than ten analogues and provided a new group of bioactive compounds for the research in future.

## Figures and Tables

**Table 1:** ^1^H NMR (600 MHz) and ^13^C NMR (150 MHz) data of compounds **1** and **2** in DMSO-*d*_6_.

Position	1		2	
	*δ*_H_ (*J* in Hz)	*δ*_C_, Type	*δ*_H_ (*J* in Hz)	*δ*_C_, Type
-NH	12.44 s	-	12.40 s	-
2	-	136.2 C	-	139.7, C
3	7.31 s	112.9 CH	6.31 s	106.1, CH
3a	-	134.6 C	-	132.6, C
4	-	174.6 C	-	174.3, C
4a	-	126.8 C	-	126.5, C
-NH	12.44 s	-	12.20 s	-
6	-	144.4 C	-	144.0, C
7	6.38 s	104.9 CH	6.29 s	104.9, CH
7a	-	132.1 C	-	131.8, C
8	-	173.2 C	-	174.5, C
8a	-	125.9 C	-	126.4, C
1′	-	195.6 C	3.85 d (7.7)	82.3, CH
2′	3.48 m	35.5 CH	2.02 m	33.1, CH
3′	1.09 (d, 6.8)	19.3 CH_3_	0.72 d (6.8)	19.0, CH_3_
4′	1.09 (d, 6.8)	19.2 CH_3_	0.92 d (6.6)	19.3, CH_3_
5′			3.12 s	56.9, CH_3_
1″	4.27 (dd, 5.1, 6.6)	71.6 CH	4.24 d (6.6)	72.0, CH
2″	1.92 m	33.8 CH	1.92 m	34.8, CH
3″	0.77 (d, 6.8)	18.8 CH_3_	0.76 d (6.8)	19.2, CH_3_
4″	0.86 (d, 6.7)	18.1 CH_3_	0.87 d (6.7)	18.5, CH_3_
1″-OH	5.27 (d, 5.2)	-	5.19 s	-

**Table 2:** IC_50_ Values (μM) of the cytotoxicities of compounds **1**–**6**.

Compounds	IC_50_ (μM)	
	A549	HepG2
**1**	133.45	111.15
**2**	98.82	84.69
**3**	62.67	57.39
**4**	0.117	0.212
**5**	>150	>150
**6**	42.53	43.87
adriamycin	3.5	1.2

**Table 3:** EC_50_ and non-toxic concentrations of compounds **1**–**6**.

Compounds	EC_50_ (μM)	Non-Toxic Concentrations (μM)
**1**	20.3	3.04–304.87
**2**	30.7	2.90–190.69
**3**	20.6	2.79–279.32
**4**	1.0	0.02–0.12
**5**	121.0	3.18–318.47
**6**	142.5	2.41–241.54
resveratrol	1.6	0.43–109.52

## Data Availability

Data are contained within the article and Supplementary Materials. Samples of the isolated compounds are not available from the authors, due to consumption for structure elucidation and bioassays.

## Supplementary Materials

The following supporting information can be downloaded at: https://www.mdpi.com/article/10.3390/molecules28237847/s1, Figure S1: HR-ESI-MS spectrum of compound **1**; Figure S2: ^1^H NMR (DMSO-*d*_6_,600 MHz) spectrum of compound **1**; Figure S3: ^13^C NMR (DMSO-*d*_6_,150 MHz) spectrum of compound **1**; Figure S4: HSQC (DMSO-*d*_6_, 600 MHz) spectrum of compound **1**; Figure S5: HMBC (DMSO-*d*_6_, 600 MHz) spectrum of compound **1**; Figure S6: ^1^H-^1^H COSY (DMSO-*d*_6_, 600 MHz) spectrum of compound **1**; Figure S7: NOESY (DMSO-*d*_6_, 600 MHz) spectrum of compound **1**; Figure S8: HR-ESI-MS spectrum of compound **1a**; Figure S9: ^1^H NMR (DMSO-*d*_6_, 600 MHz) spectrum of compound **1a**; Figure S10: ^1^H-^1^H COSY (DMSO-*d*_6_, 600 MHz) spectrum of compound **1a**; Figure S11: HR-ESI-MS spectrum of compound **1b**; Figure S12: ^1^H NMR (DMSO-*d*_6_, 600 MHz) spectrum of compound **1b**; Figure S13: ^1^H-^1^H COSY (DMSO-*d*_6_, 600 MHz) spectrum of compound **1b**; Figure S14: HR-ESI-MS spectrum of compound **2**; Figure S15: ^1^H NMR (DMSO-*d*_6_, 600 MHz) spectrum of compound **2**; Figure S16: ^13^C NMR (DMSO-*d*_6_,150 MHz) spectrum of compound **2**; Figure S17: HMBC (DMSO-*d*_6_, 600 MHz) spectrum of compound **2**; Figure S18: NOESY (DMSO-*d*_6_, 600 MHz) spectrum of compound **2**; Figure S19: HSQC (DMSO-*d*_6_, 600 MHz) spectrum of compound **2**; Figure S20: ^1^H-^1^H COSY (DMSO-*d*_6_, 600 MHz) spectrum of compound **2**; Figure S21: Dose-response curves of compounds **1**–**6** against A549 cells; Figure S22: Dose-response curves of compounds **1**–**6** against Hep G2 cells.
