# Supplementary material for: New Dipyrroloquinones from a Plant-Derived Endophytic Fungus Talaromyces sp
Source: Molecules. 2023 Nov 29;28(23):7847. doi: 10.3390/molecules28237847 (PMC10708468; doi:10.3390/molecules28237847)
Supplement: Supplementary file 1 [file molecules-28-07847-s001.zip › molecules-2703690-supplementary.pdf]

New dipyrroloquinones from a plant-derived endophytic fungus *Talaromyces* sp.

Dan-Dan Zhang[1,†], Xiao-Qing Wang[1,†], Bo Liu[1], Shu-Hui Li[1], Yan-Lei Wang[1], Tao Guo[2], Yi Sun[1,*]

[1] Institute of Chinese Materia Medica, China Academy of Chinese Medical Sciences, Beijing100010, China; z2531817909@163.com (D.-D.Z.); lubov_lb@126.com (B.L.); xiaoqw1307@outlook.com (X.-Q.W.); l17744140302@163.com (S.-H.L.); w15739539626@163.com (Y.-L.W.);

[2] Henan engineering research center of Medicinal and Edible Chinese Medicine Technology, Henan university of Chinese Medicine, Zhengzhou 450046, China.

* Correspondence: ysun@icmm.ac.cn (Y.S.). Tel.: +86-10-6403-2656 (Y.S.); † These two authors contribute to this work equally

Contents

Figure S1. HR-ESI-MS spectrum of compound 1.......... 3
Figure S2. ^{1}H NMR (DMSO-d_6, 600 MHz) spectrum of compound 1. 3
Figure S3. ^{13}C NMR (DMSO-d_6,150 MHz) spectrum of compound 1.......... 4
Figure S4. HSQC (DMSO-d6, 600 MHz) spectrum of compound 1. 4
Figure S5. HMBC (DMSO-d_6, 600 MHz) spectrum of compound 1.......... 5
Figure S6. ^{1}H-^{1}H COSY (DMSO-d_6, 600 MHz) spectrum of compound 1. 5
Figure S7. NOESY (DMSO-d_6, 600 MHz) spectrum of compound 1. 6
Figure S8. HR-ESI-MS spectrum of compound 1a. 6
Figure S9. HR-ESI-MS spectrum of compound 1b. 7
Figure S10. ^{1}H NMR (DMSO-d_6,600 MHz) spectrum of compound 1a.......... 7
Figure S11. ^{1}H-^{1}H COSY (DMSO-d_6, 600 MHz) spectrum of compound 1a.......... 8
Figure S12. ^{1}H NMR (DMSO-d_6,600 MHz) spectrum of compound 1b.......... 8
Figure S13. ^{1}H-^{1}H COSY (DMSO-d_6, 600 MHz) spectrum of compound 1b. 9
Figure S14. HR-ESI-MS spectrum of compound 2. 10
Figure S15. ^{1}H NMR (DMSO-d_6,600 MHz) spectrum of compound 2. 10
Figure S16. ^{13}C NMR (DMSO-d_6,150 MHz) spectrum of compound 2. 10
Figure S17. HMBC (DMSO-d_6, 600 MHz) spectrum of compound 2. 11
Figure S18. NOESY (DMSO-d_6, 600 MHz) spectrum of compound 2. 11
Figure S19. HSQC (DMSO-d_6, 600 MHz) spectrum of compound 2. 12
Figure S20. ^{1}H-^{1}H COSY (DMSO-d_6, 600 MHz) spectrum of compound 2. 12
Figure S21. Dose-response curves of compounds 1-6 against A549 cells.......... 9
Figure S22. Dose-response curves of compounds 1-6 against Hep G2 cells. 9

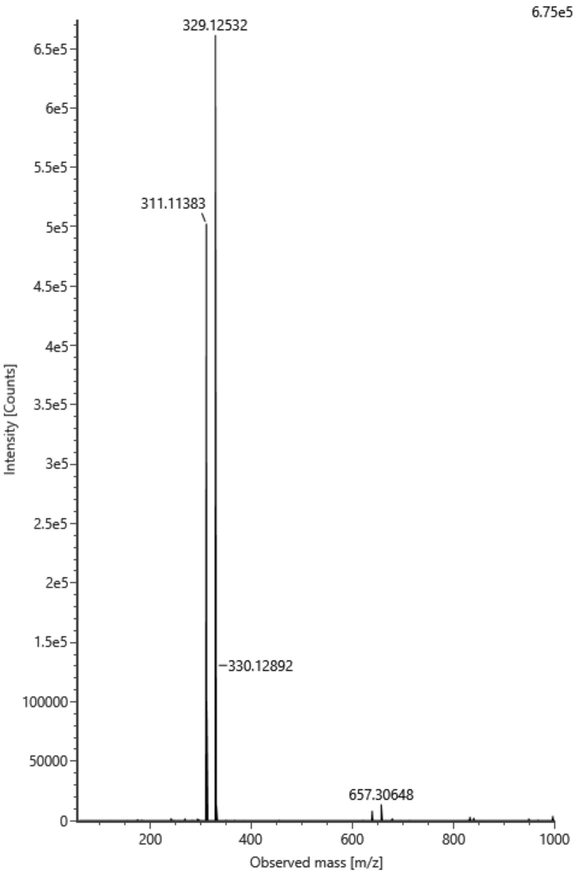

Figure S1. HR-ESI-MS spectrum of compound **1**.

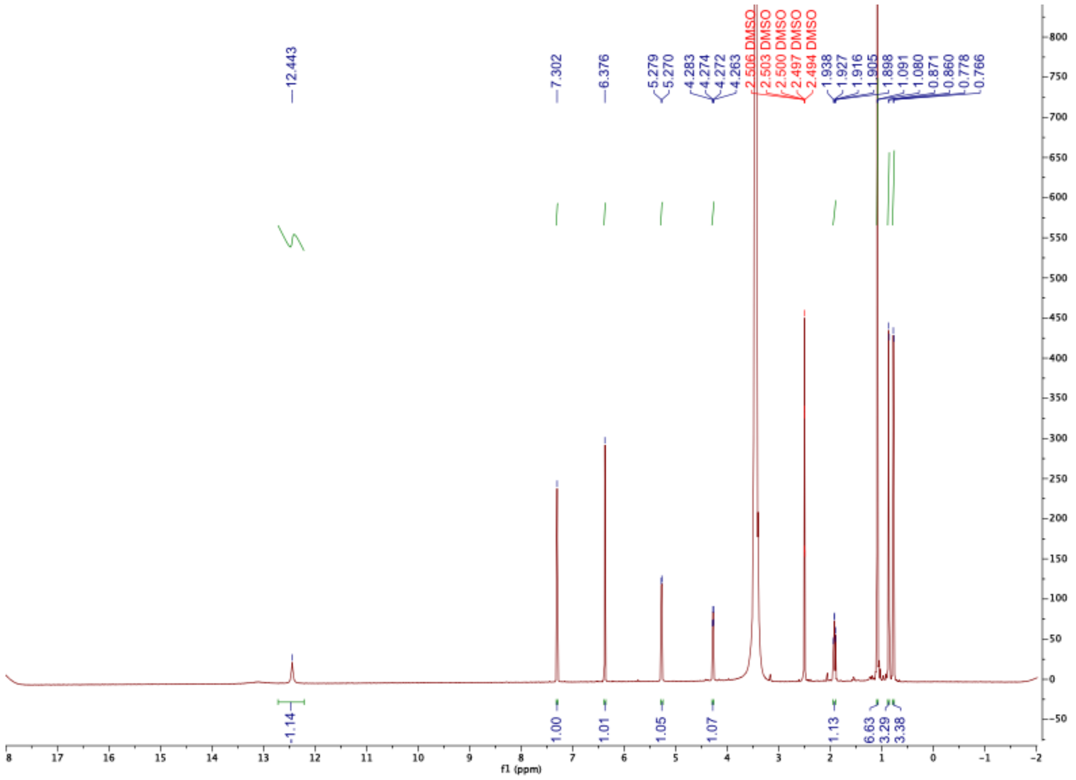

Figure S2. ^{1}H NMR (DMSO-d_6,600 MHz) spectrum of compound **1**.

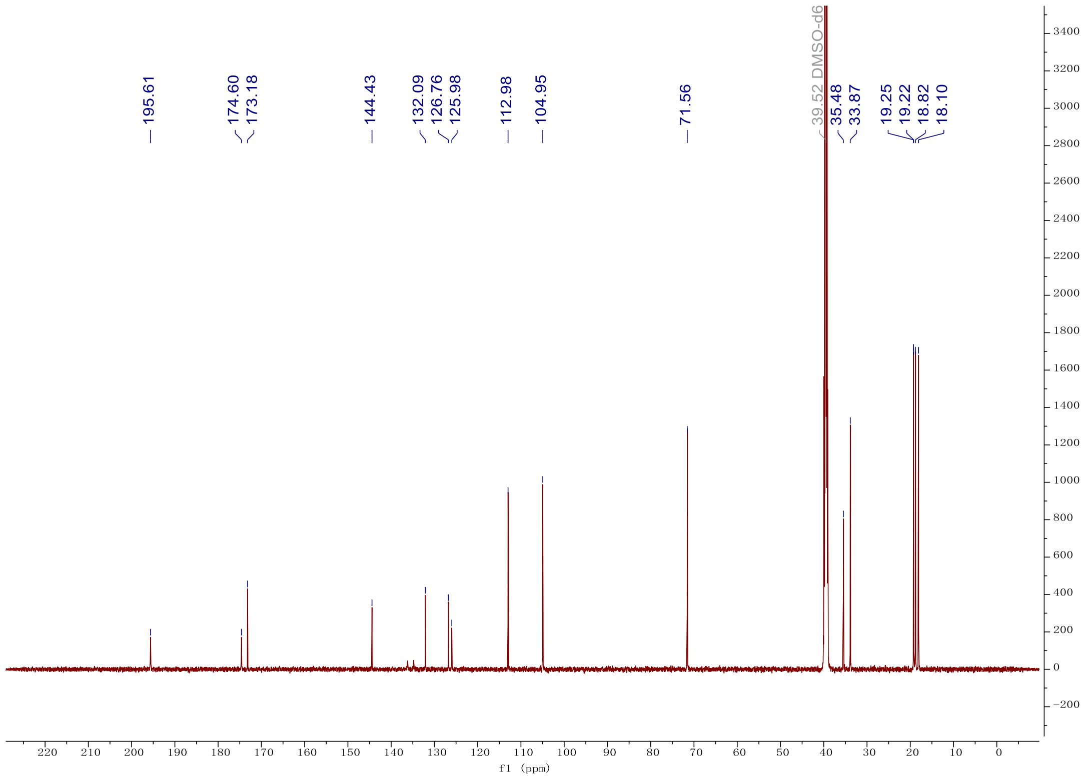

Figure S3. ^{13}C NMR (DMSO-d_6,150 MHz) spectrum of compound **1**.

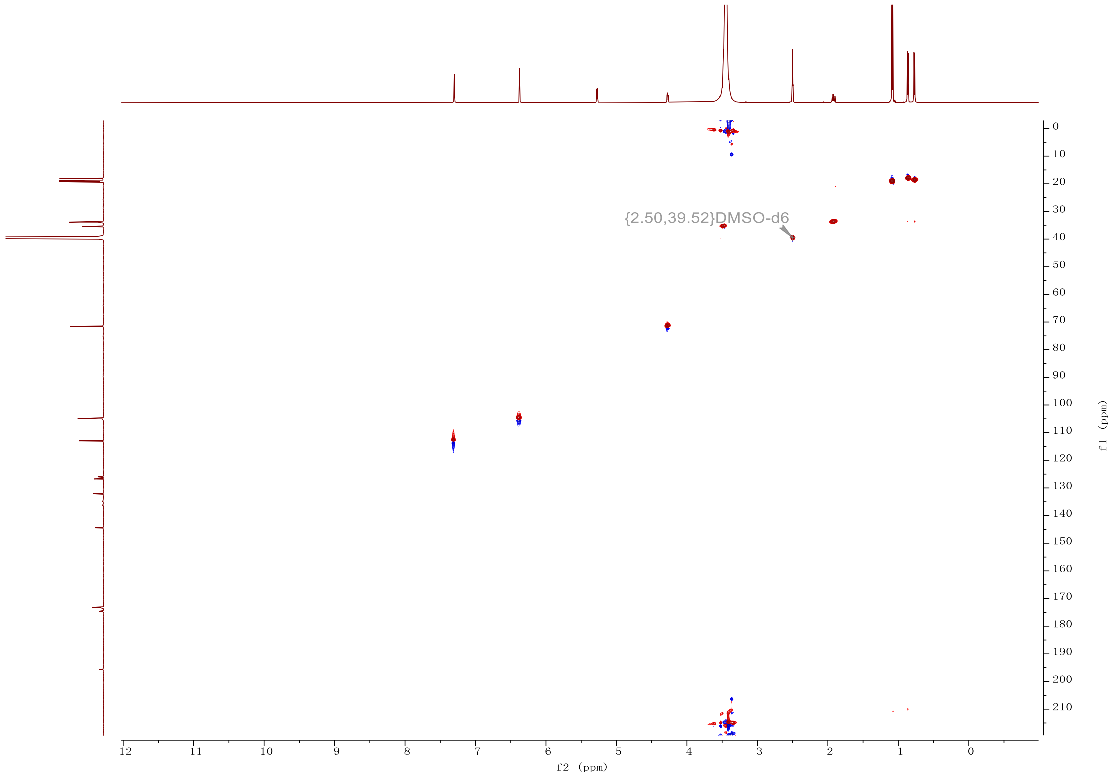

Figure S4. HSQC (DMSO-d6, 600 MHz) spectrum of compound **1**.

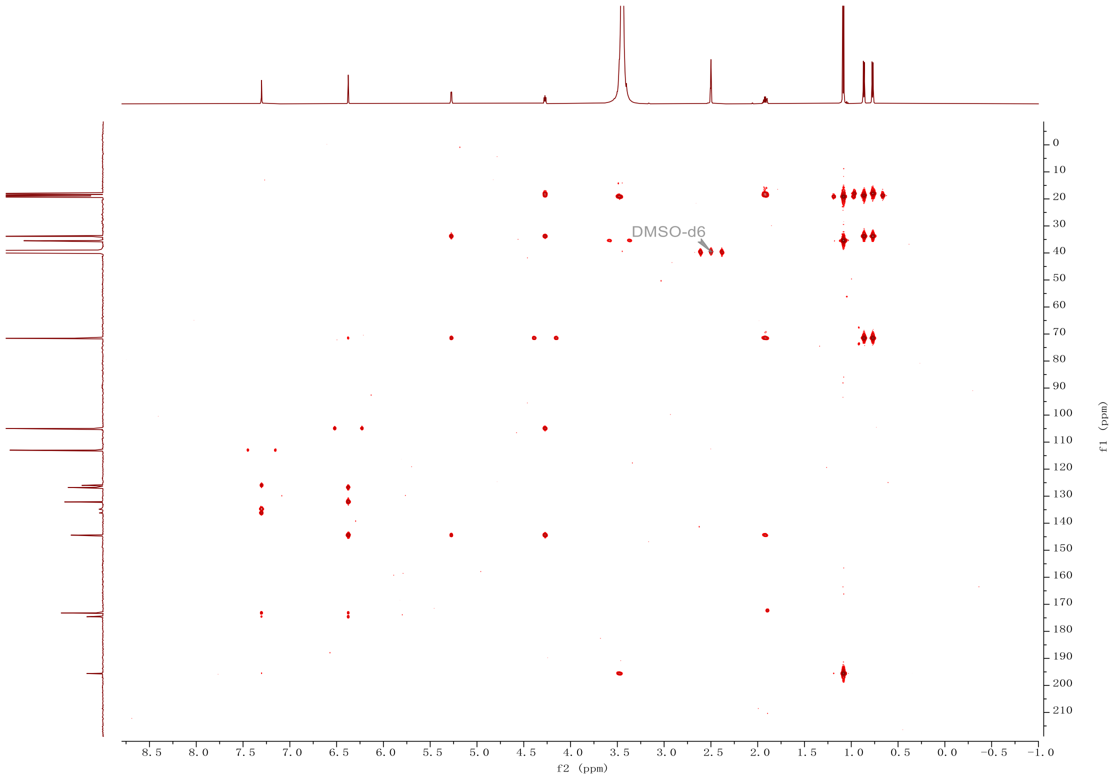

Figure S5. HMBC (DMSO-*d*6, 600 MHz) spectrum of compound **1**.

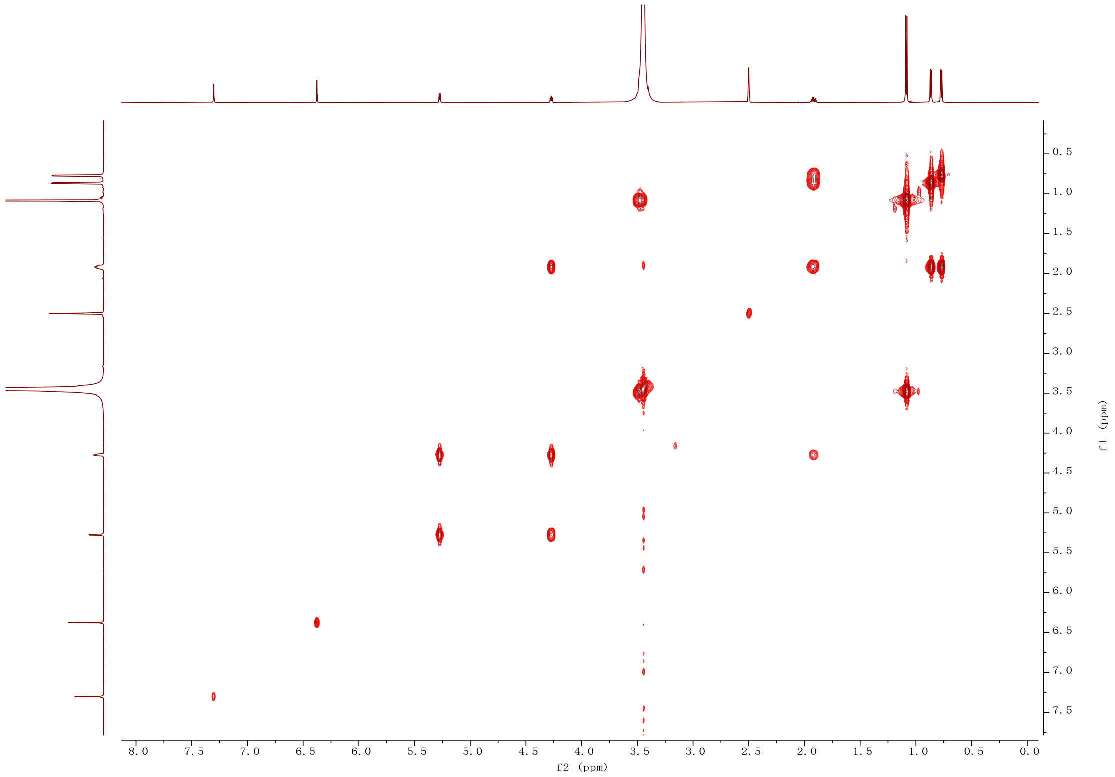

Figure S6. ^{1}H-^{1}H COSY (DMSO-*d*6, 600 MHz) spectrum of compound **1**.

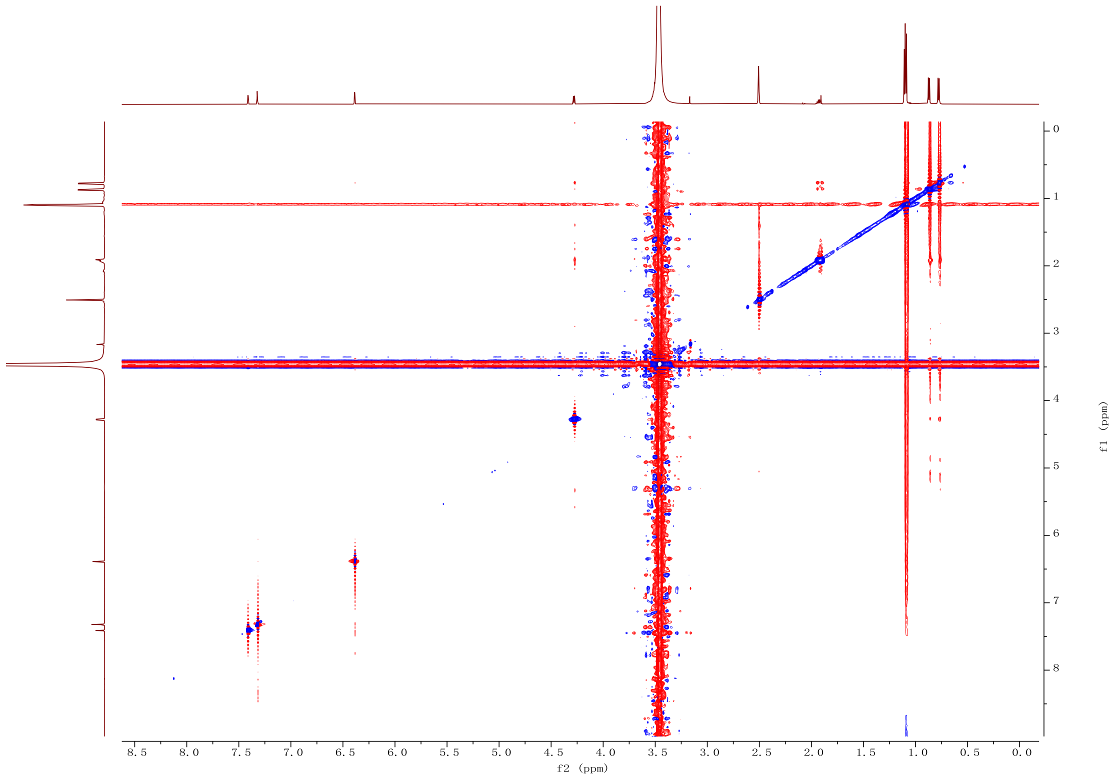

Figure S7. NOESY (DMSO-d_6, 600 MHz) spectrum of compound **1**.

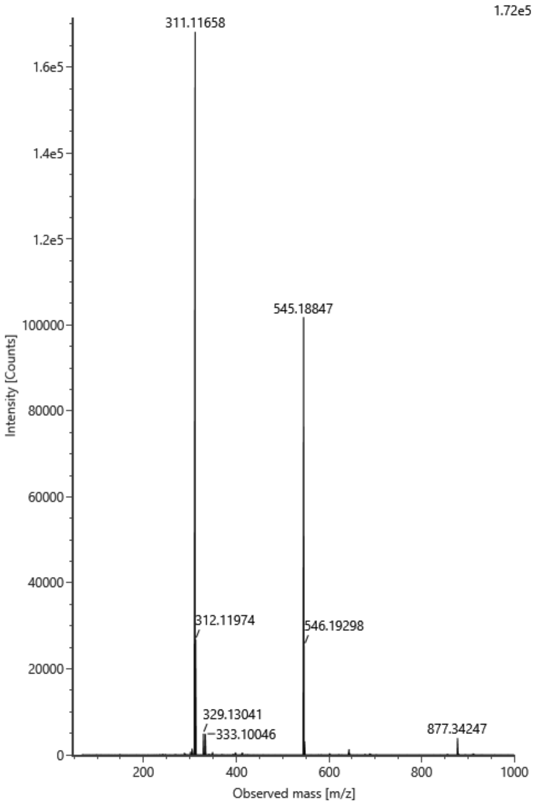

Figure S8. HR-ESI-MS spectrum of compound **1a**.

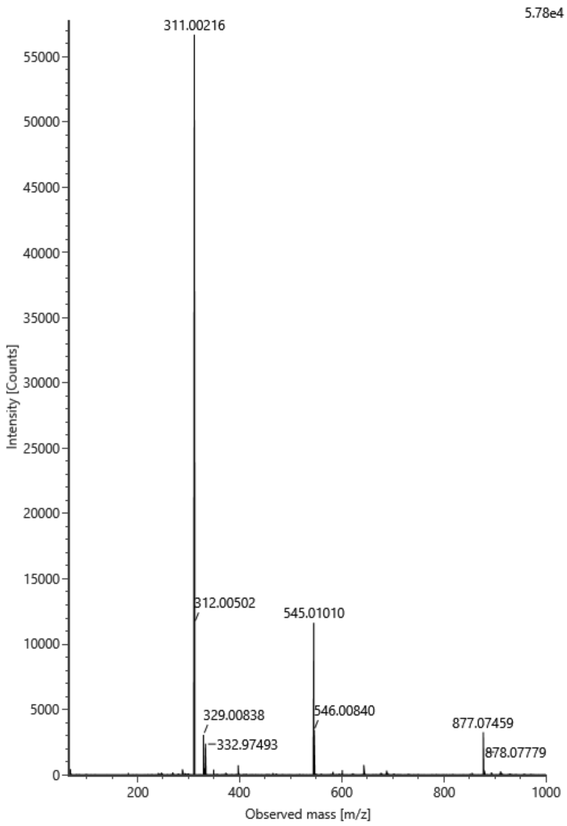

Figure S9. HR-ESI-MS spectrum of compound **1b**.

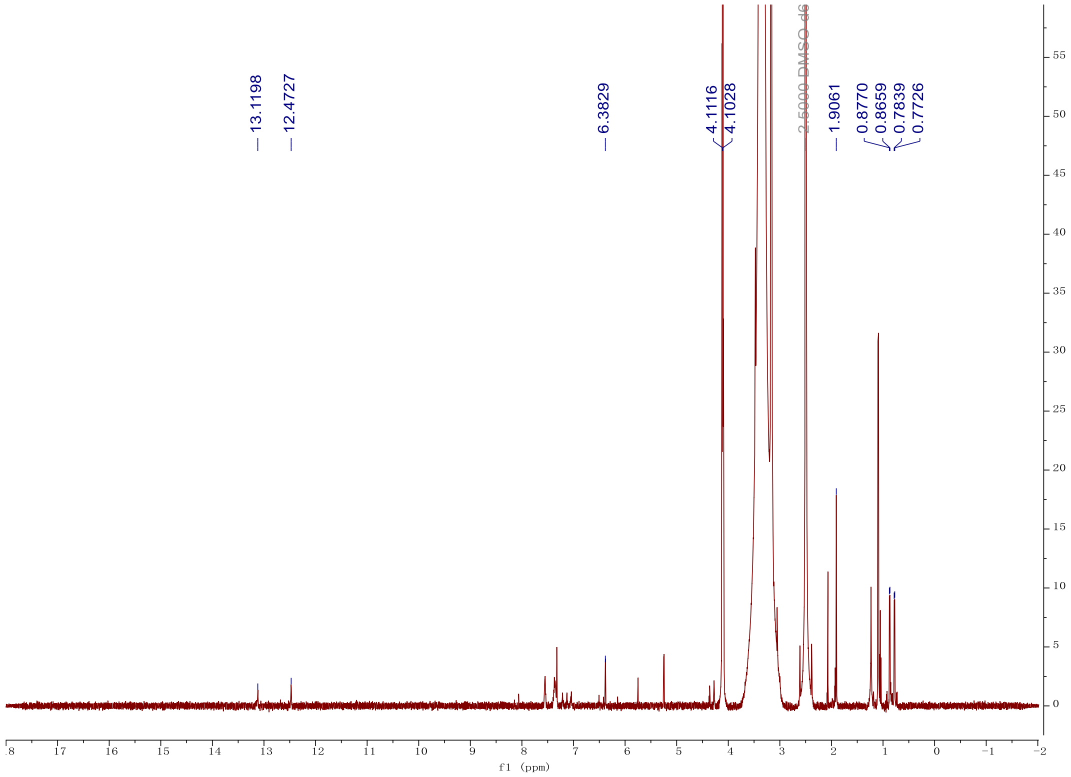

Figure S10. ^{1}H NMR (DMSO-d_6,600 MHz) spectrum of compound **1a**.

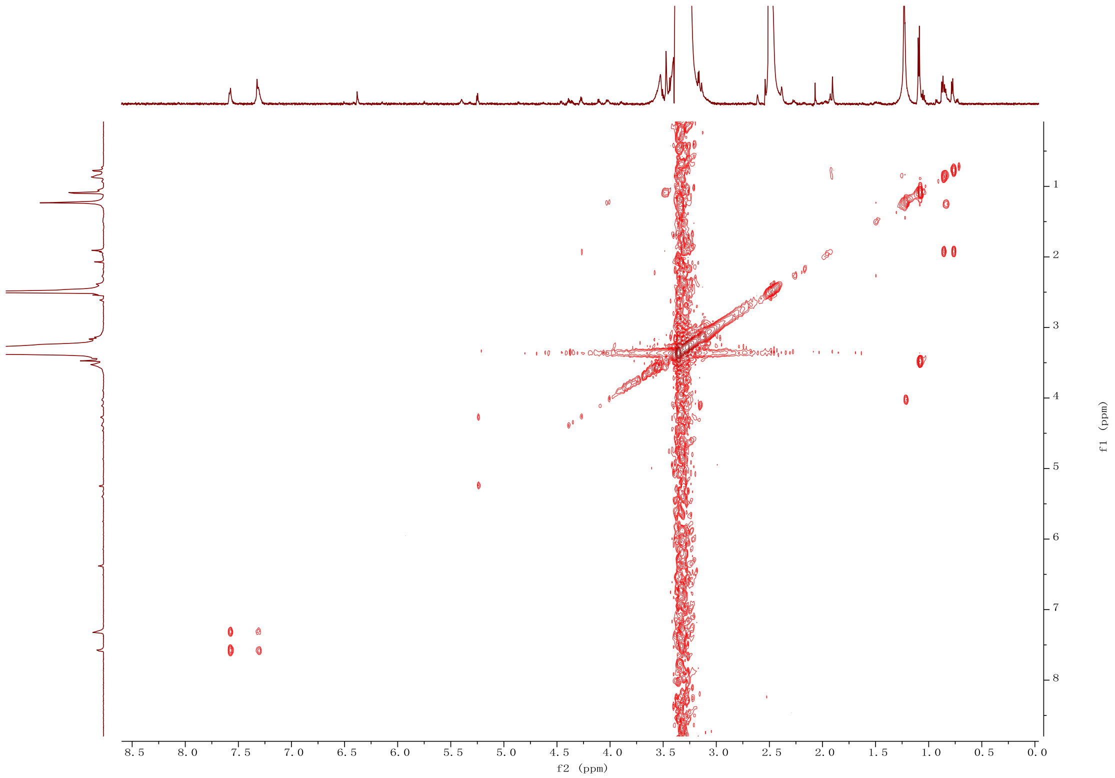

Figure S11. ^{1}H-^{1}H COSY (DMSO-d_6, 600 MHz) spectrum of compound **1a**.

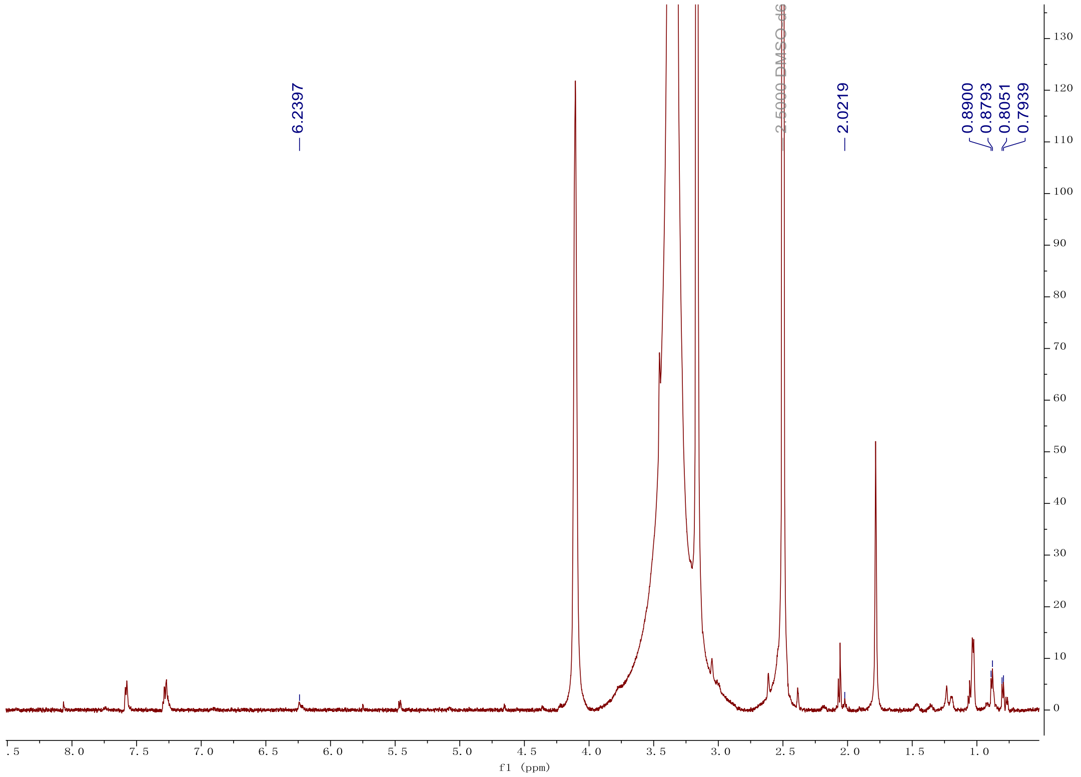

Figure S12. ^{1}H NMR (DMSO-d_6,600 MHz) spectrum of compound **1b**.

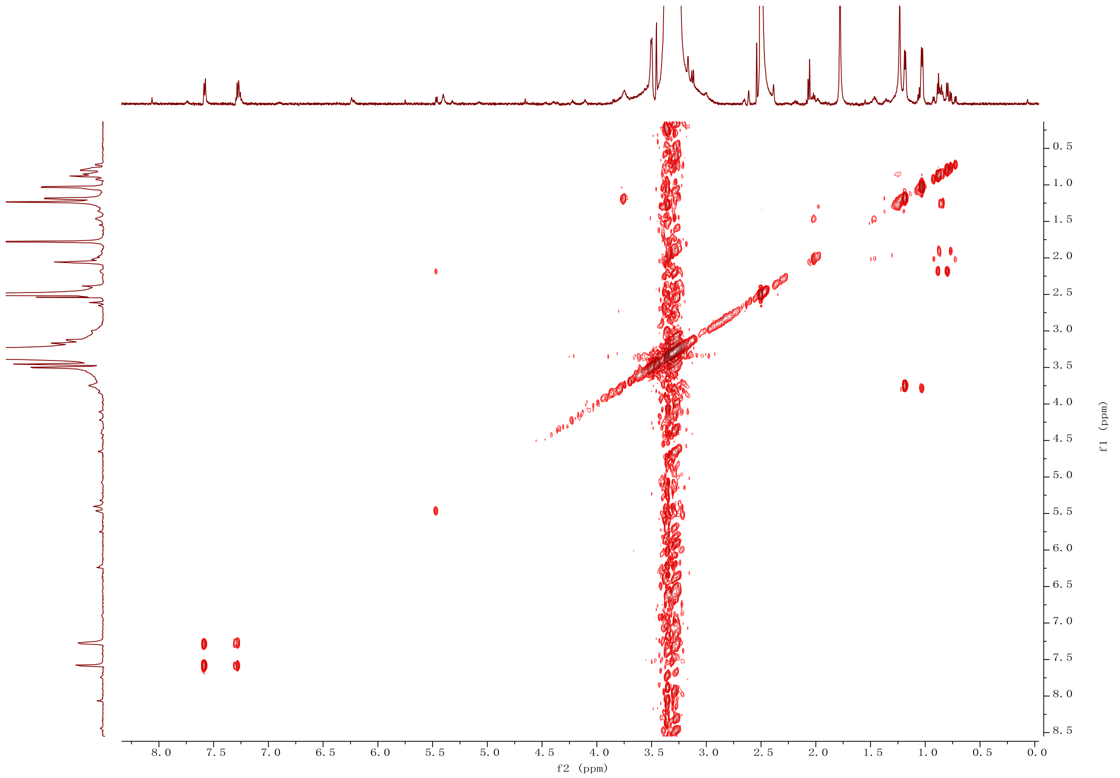

Figure S13. ^{1}H-^{1}H COSY (DMSO-d_6, 600 MHz) spectrum of compound **1b**.

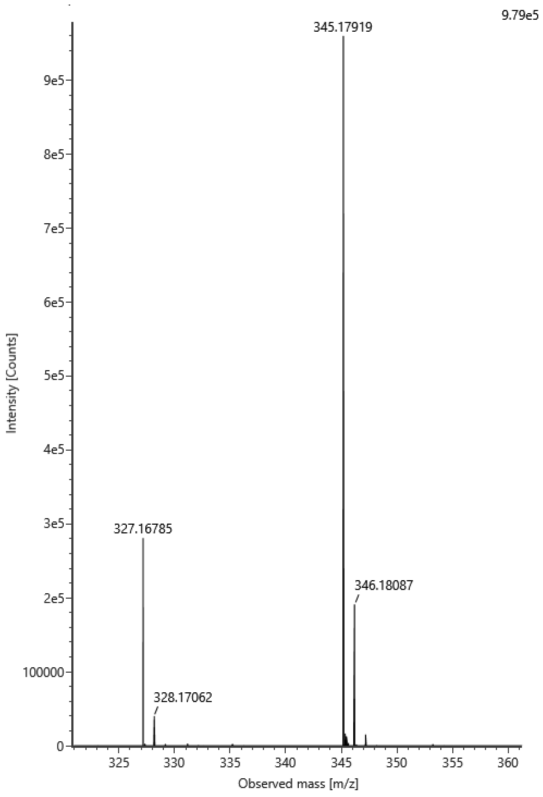

Figure S14. HR-ESI-MS spectrum of compound **2**.

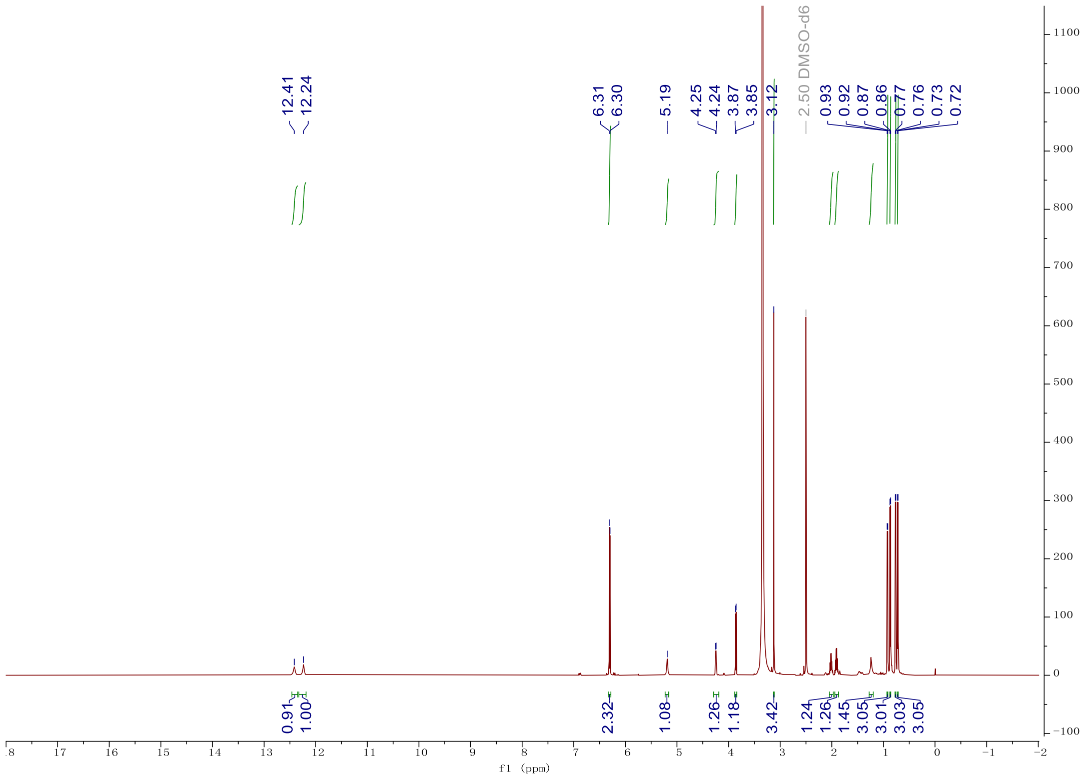

Figure S15. ^{1}H NMR (DMSO-d_6,600 MHz) spectrum of compound **2**.

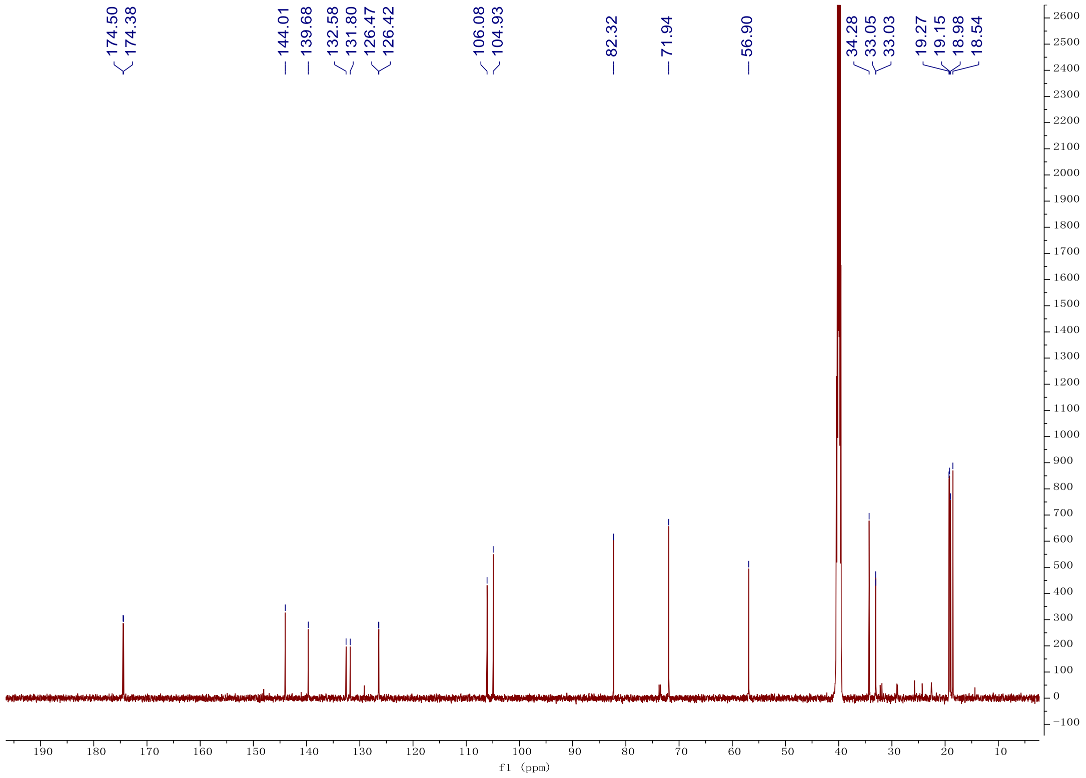

Figure S16. ^{13}C NMR (DMSO-d_6,150 MHz) spectrum of compound **2**.

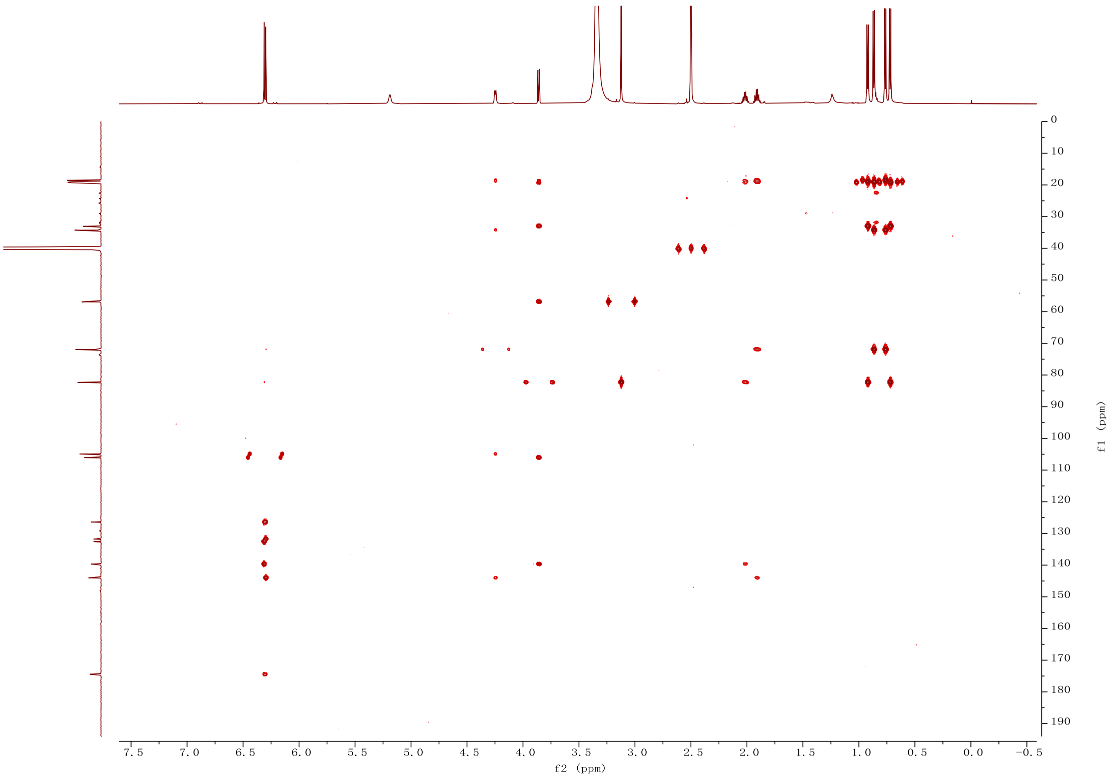

Figure S17. HMBC (DMSO-d_6, 600 MHz) spectrum of compound **2**.

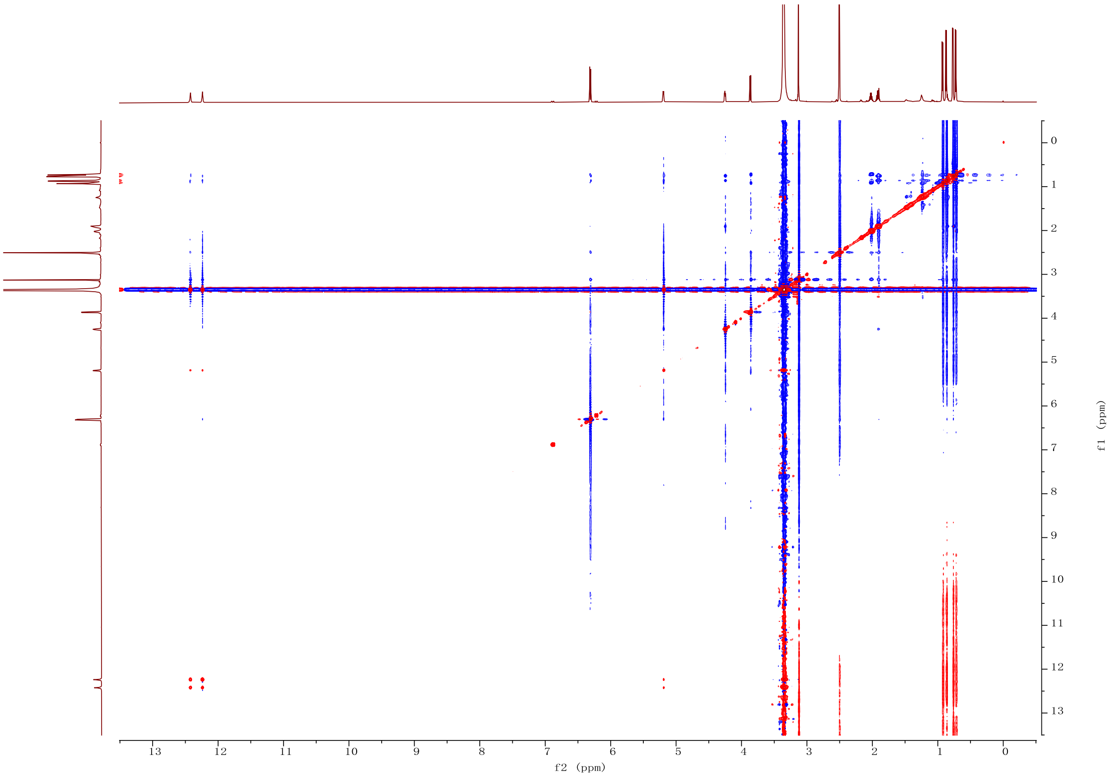

Figure S18. NOESY (DMSO-d_6, 600 MHz) spectrum of compound **2**.

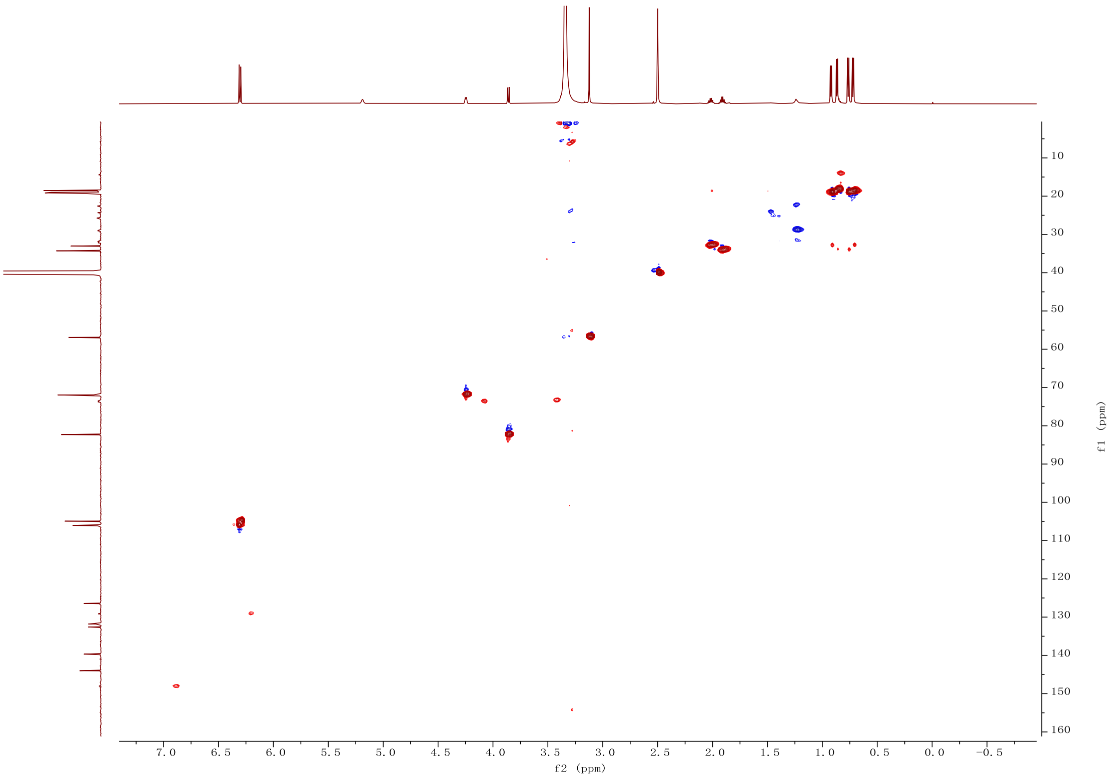

Figure S19. HSQC (DMSO-*d*6, 600 MHz) spectrum of compound **2**.

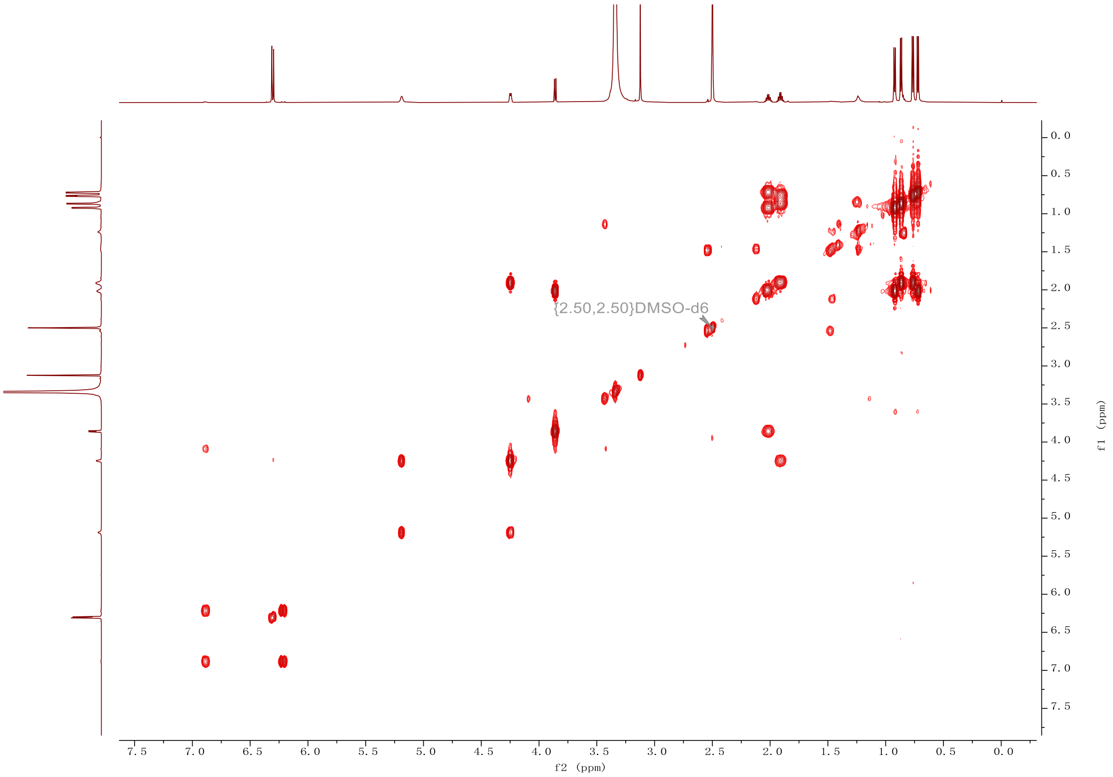

Figure S20. ^{1}H-^{1}H COSY (DMSO-*d*6, 600 MHz) spectrum of compound **2**.

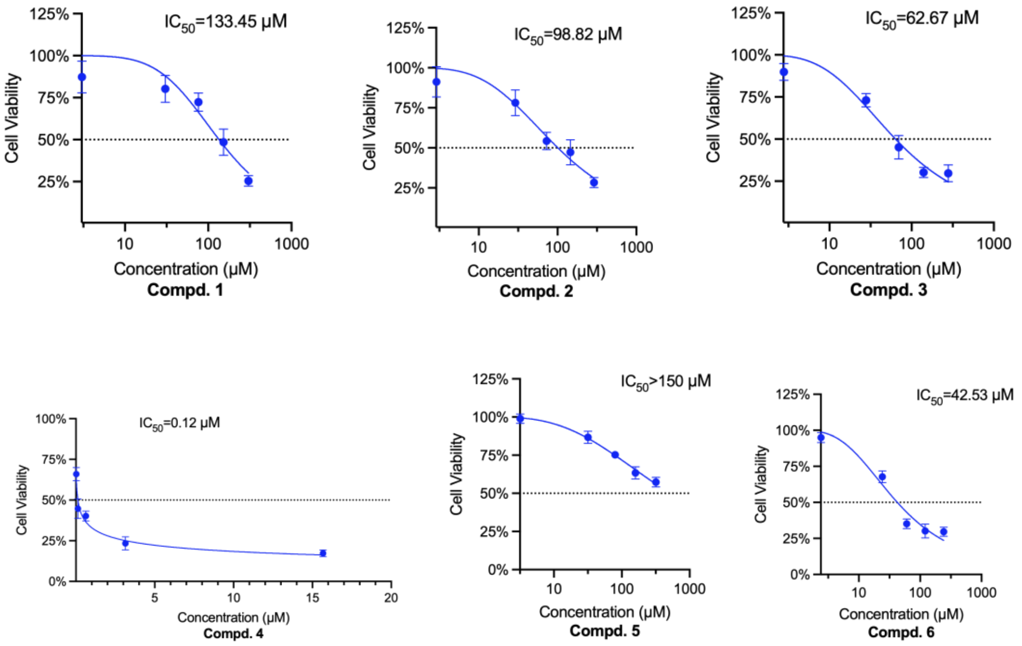

Figure S21. Dose-response curves of compounds **1-6** against A549 cells.

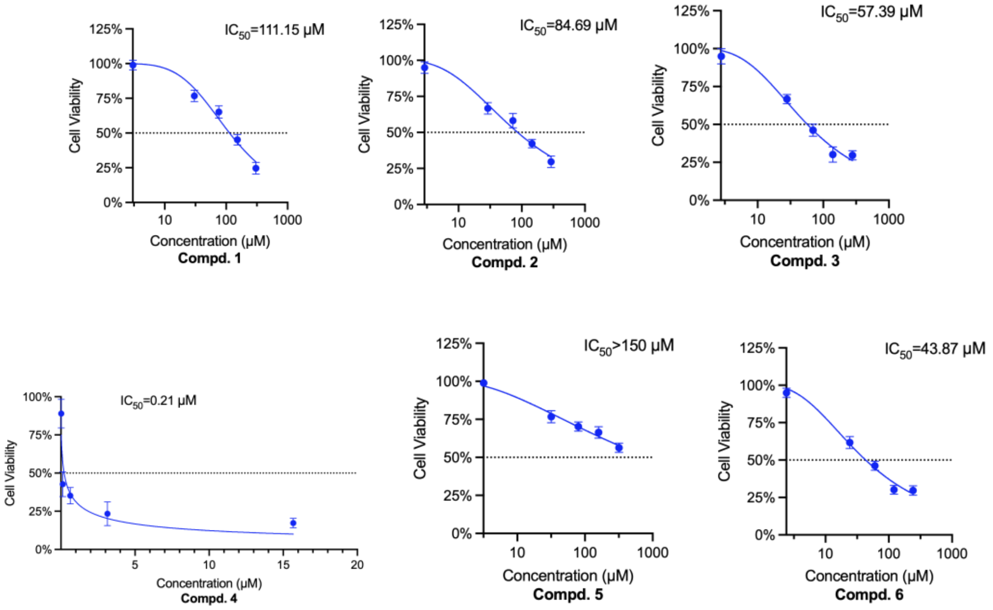

Figure S22. Dose-response curves of compounds **1-6** against Hep G2 cells.
